# Tragedy of the commons in the chemostat

**DOI:** 10.1371/journal.pone.0186119

**Published:** 2017-12-20

**Authors:** Martin Schuster, Eric Foxall, David Finch, Hal Smith, Patrick De Leenheer

**Affiliations:** 1 Department of Microbiology, Oregon State University, Corvallis, OR, United States of America; 2 School of Mathematical & Statistical Sciences, Arizona State University, Tempe, AZ, United States of America; 3 Department of Mathematics, Oregon State University, Corvallis, OR, United States of America; King’s College London, UNITED KINGDOM

## Abstract

We present a proof of principle for the phenomenon of the tragedy of the commons that is at the center of many theories on the evolution of cooperation. Whereas the tragedy is commonly set in a game theoretical context, and attributed to an underlying Prisoner’s Dilemma, we take an alternative approach based on basic mechanistic principles of species growth that does not rely on the specification of payoffs which may be difficult to determine in practice. We establish the tragedy in the context of a general chemostat model with two species, the cooperator and the cheater. Both species have the same growth rate function and yield constant, but the cooperator allocates a portion of the nutrient uptake towards the production of a public good -the “Commons” in the Tragedy- which is needed to digest the externally supplied nutrient. The cheater on the other hand does not produce this enzyme, and allocates all nutrient uptake towards its own growth. We prove that when the cheater is present initially, both the cooperator and the cheater will eventually go extinct, hereby confirming the occurrence of the tragedy. We also show that without the cheater, the cooperator can survive indefinitely, provided that at least a low level of public good or processed nutrient is available initially. Our results provide a predictive framework for the analysis of cooperator-cheater dynamics in a powerful model system of experimental evolution.

## Introduction

Cooperative behavior abound across all domains of life, from animals to microbes [1, 2]. Yet, it can only evolve and be maintained under specific conditions [3–7]. Why would an individual carry out a costly behavior for the benefit of the group? Cheaters that reap the benefits of cooperation without paying the costs would gain a competitive advantage and invade the population. This conflict of interest between the individual and the group is also known as the “tragedy of the commons” described by Hardin [8]. To illustrate the tragedy, Hardin considers a scenario first sketched by Lloyd more than 100 years earlier [9], a pasture that is shared by herdsmen. It is in each herdsman’s best interest to add additional cattle to the pasture, because he gains the profits from individual cattle sales, but shares the costs of overgrazing with all other herdsmen. This behavior is pursued until, ultimately, the commons is destroyed to the detriment of all.

The problem of cooperation has received considerable attention in the microbial realm [2, 10, 11]. Many microbes perform cooperative behaviors such as biofilm formation, virulence, and collective nutrient acquisition. Often, these behaviors are accomplished by secreted products referred to as public goods [2, 7]. Public goods are costly to produce for the individual but provide a collective benefit to the local group. They include extracellular enzymes that degrade complex food sources, siderophores that scavenge iron from the environment, and secreted toxins and antibiotics that harm other cells. It has been shown in several microbial systems that public goods can be shared within a population of cells, benefitting cells other than the focal producer [12–16]. For example, when the bacterium *Pseudomonas aeruginosa* is grown on a proteinaceous substrate, mutants deficient in protease secretion enrich in co-culture with the wild-type parent [12, 15]. These non-producing strains are termed obligate cheaters: They cannot grow by themselves, but they have a relative growth advantage in mixed cultures with cooperators. Because cheater enrichment inevitably imposes a burden on the population, the expected outcome is a collapse of the population [17]. This outcome been shown experimentally in a few cases [14, 18, 19]. Often, however, cooperative behaviors are stably maintained and hence, the focus has largely been on mechanisms that avoid a tragedy of the commons. These include spatial structure, population fragmentation, pleiotropic regulation of cooperative traits, the stability and prudent regulation of public goods to minimize costs, and nonsocial adaptation to new environments [20–30].

To our knowledge, the notion that obligate cheating behavior constitutes a tragedy of the commons and leads to population collapse has not been mathematically proven. Here, we consider the dynamics between cooperators and obligate cheaters in a continuous culture system. Continuous cultures or chemostats enable microbial culturing at a specified density and growth rate through the constant dilution of the culture with fresh growth medium [31]. There is an extensive mathematical theory that describes population dynamics in the chemostat [31]. We prove that obligate cheaters inevitably increase in frequency until cooperation via public goods is no longer sustainable, eventually leading to wash-out and population collapse. We also show that the dynamics of the cooperators in the absence of cheaters exhibits bistability: Depending on the initial condition of the system, cooperators will either eventually persist, or go extinct. In summary, populations solely comprised of cooperators have a chance to persist, but they are doomed whenever cheaters arise, even at low initial frequency.

## Results

We propose a chemostat model where *S* denotes the concentration of the unprocessed nutrient, *P* of the processed nutrient, *E* of the enzyme and *X*_1_ is the concentration of the cooperator who produces an enzyme required for nutrient processing, and *X*_2_ of the cheater who does not produce the enzyme. Following standard chemostat modeling ideas [31], the mass-balance equations for these variables are as follows:

$$\begin{array}{ccc} \frac{dS}{dt}\left(t\right) & = & D\left(t\right)(S^{0}\left(t\right)-S)-G\left(E,S\right) \end{array}$$ (1)

$$\begin{array}{ccc} \frac{dP}{dt}\left(t\right) & = & G\left(E,S\right)-\frac{1}{\gamma }\left(X_{1}+X_{2}\right)F\left(P\right)-D\left(t\right)P \end{array}$$ (2)

$$\begin{array}{ccc} \frac{dE}{dt}\left(t\right) & = & \left(1-q\right)X_{1}F\left(P\right)-D\left(t\right)E \end{array}$$ (3)

$$\begin{array}{ccc} \frac{dX_{1}}{dt}\left(t\right) & = & X_{1}\left(qF\left(P\right)-D\left(t\right)\right) \end{array}$$ (4)

$$\begin{array}{ccc} \frac{dX_{2}}{dt}\left(t\right) & = & X_{2}\left(F\left(P\right)-D\left(t\right)\right) \end{array}$$ (5)

**Fig 1:**
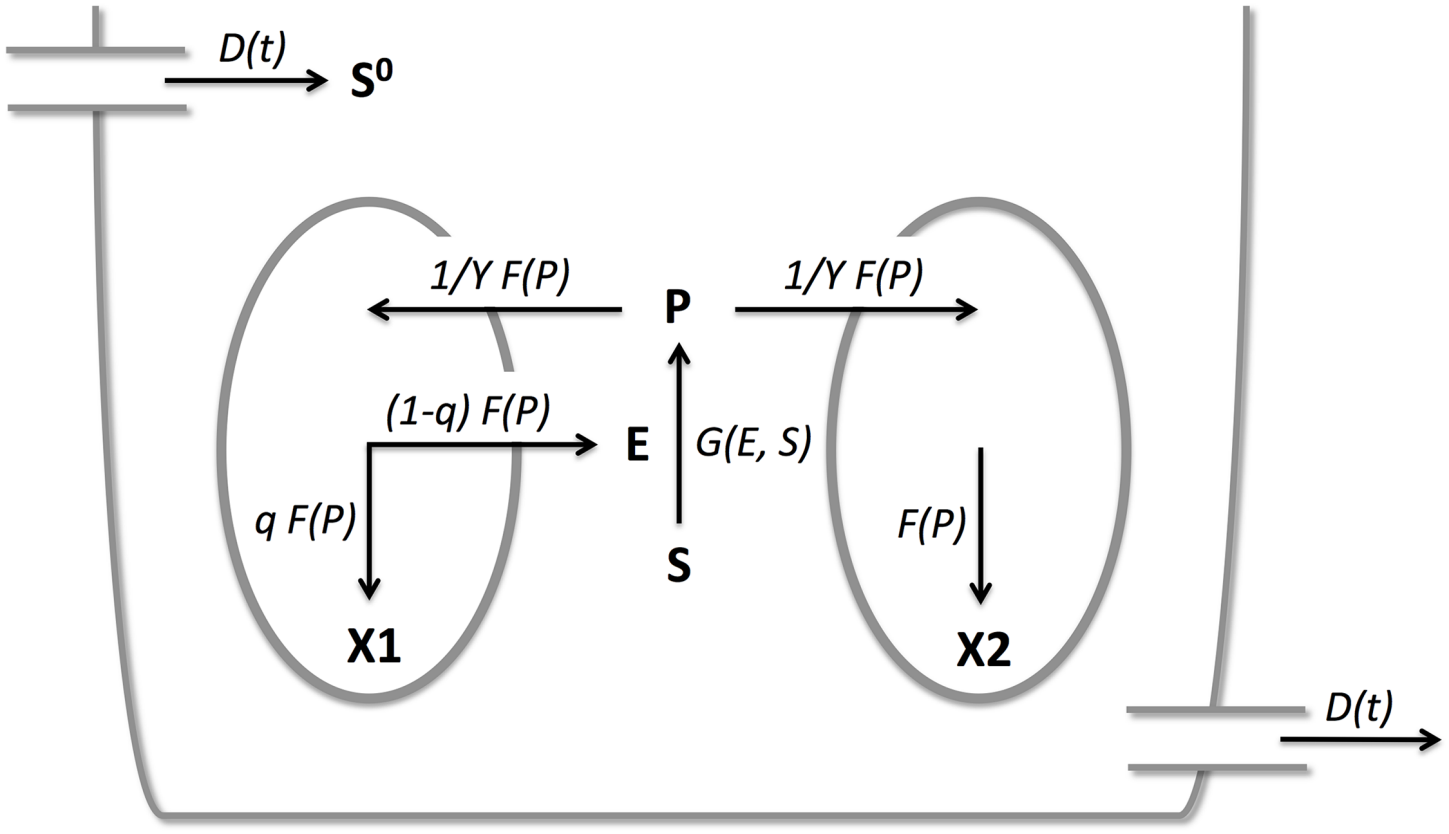
Cartoon of the chemostat with two competing cell types. Species are indicated in bold and rates are indicated in italics. *X*_1_, cooperator; *X*_2_, cheater; *S*, nutrient substrate; *S*^0^, unprocessed nutrient substrate in inflow; *P*, processed nutrient; *E*, enzyme; *D*(*t*), dilution rate, 1/*yF*(*P*), per capita nutrient consumption rate; *F*(*P*), growth rate; *q* and 1 − *q*, proportions of nutrient allocated towards growth and enzyme production, respectively.

The operating conditions of the chemostat may fluctuate in time, and they are characterized by *D*(*t*), the dilution rate, and *S*^0^(*t*), the concentration of the unprocessed nutrient at the inflow. Both are non-negative functions of time, and additional assumptions for these functions will be introduced below. Unprocessed nutrient is converted into processed nutrient by means of the enzyme. Processed nutrient is produced at rate *G*(*E*, *S*). The per capita consumption rate of processed nutrient by both species is the same, and denoted by $\frac{1}{\gamma }F\left(P\right)$, where *γ* is the yield of this process, which is also assumed to be the same for both species. The cooperator allocates a proportion *q*, a fixed value in (0, 1), of the processed nutrient it has consumed, towards its own growth. The remaining fraction (1 − *q*) goes towards the production of the enzyme which is needed to process the unprocessed nutrient. The cheater allocates all processed nutrient it has taken up towards growth. A cartoon of this chemostat model is presented in Fig 1.

We make the following minimal assumptions about the functions *G* and *F*:

$$\begin{array}{ccc} & H1:G:R_{+}\times R_{+}\to R_{+}\;\text{is}\;C^{1},G\left(0,S\right)=G\left(E,0\right)=0\;\text{for}\;\text{all}\;E\geq 0\;\text{and}\;S\geq 0,\;\text{and} & \\ & F:R_{+}\to R_{+}\;\text{is}\;C^{1},\;\text{and}\;F\left(0\right)=0. \end{array}$$

This assumption merely implies that there is no conversion of unprocessed nutrient into processed nutrient, when the enzyme or the unprocessed nutrient is missing; similarly there is no growth of either species, or of the enzyme, when the processed nutrient is missing.

For the dilution rate *D*(*t*), and input nutrient concentration *S*^0^(*t*), we assume the following:

$$\begin{array}{ccc} & H2:\text{The}\;\text{functions}\;D\left(t\right)\;\text{and}\;S^{0}\left(t\right)\;\text{are}\;\text{continuous}\;\text{for}\;\text{all}\;t\geq 0,\;\text{and}\;\text{there}\;\text{exist}\;\text{positive} & \\ & \text{bounds}\;\underline{D}\;\text{and}\;\bar{D}\;\text{such}\;\text{that}\;\underline{D}\leq D\left(t\right)\leq \bar{D}\;\text{for}\;\text{all}\;t\geq 0,\;\text{and} & \\ & \text{positive}\;\text{bounds}\;{\underline{S}}^{0}\;\text{and}\;{\bar{S}}^{0}\;\text{such}\;\text{that}\;{\underline{S}}^{0}\leq S^{0}\left(t\right)\leq {\bar{S}}^{0}\;\text{for}\;\text{all}\;t\geq 0. \end{array}$$

Our Main Result, which is proved in the S1 Appendix, establishes the tragedy of the commons:

**Theorem 1** *Assume that* **H1** *and* **H2** *hold, and assume that the initial condition of* (1)–(5) *is such that* *X*_2_(0) > 0; *that is, the cheater is present initially. Then* (*P*(*t*), *E*(*t*), *X*_1_(*t*), *X*_2_(*t*)) → (0, 0, 0, 0) *as* *t* → ∞.

**Fig 2:**
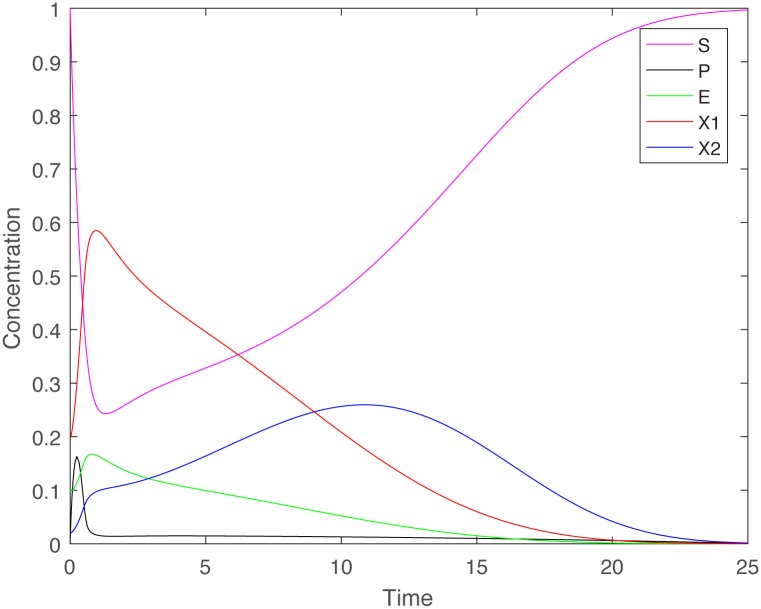
Time series of chemostat model illustrating the tragedy of the commons. Time series of the components of system (1)–(5), where *S*^0^ = 1, *D* = 1, *q* = 0.8, *γ* = 1, *G*(*E*, *S*) = *kES*, *F*(*P*) = *mP*/(*a* + *P*) with *k* = 20, *m* = 5 and *a* = 0.05. Initial data: *S*(0) = 1, *P*(0) = 0, *E*(0) = 0.1, *X*_1_(0) = 0.2, *X*_2_(0) = 0.02.

Fig 2 depicts the tragedy in case of mass action kinetics *G*(*E*, *S*) = *kES*, and Monod uptake function *F*(*P*) = *mP*/(*a* + *P*). The equations have been scaled such that *S*^0^ and *D* are both constant equal to one. Initial data are as follows: *S*(0) = 1, *P*(0) = 0, *E*(0) = 0.8, *X*_1_(0) = 0.2, *X*_2_(0) = 0.03. The cooperator peaks early and declines sharply as the cheater continues to thrive, reaching a maximum followed by a rapid decline.

We show next that the tragedy also occurs in cases where the processing of the substrate into processed nutrient proceeds in more than one step. First, let us single out the biochemical reaction taking place in model (1)–(5). Borrowing notation from (bio)chemistry, this reaction can be represented as follows:

$$\begin{array}{c} S+E\to P+E, \end{array}$$

where the reaction rate of formation of processed nutrient is *g*(*e*, *s*), expressed in rescaled variables (see S1 Appendix for the rescaling). If we would only model this process, and ignore enzyme production, inflow of substrate, and outflow of substrate, enzyme and processed nutrient, we would have the following mass balance:

$$\begin{array}{ccc} \frac{ds}{dt}\left(t\right) & = & -g(e,s)\\ \frac{de}{dt}\left(t\right) & = & 0\\ \frac{dp}{dt}\left(t\right) & = & g(e,s) \end{array}$$

Suppose now that the biochemistry describing the conversion of substrate into processed nutrient takes occurs via an intermediate step:

$$\begin{array}{c} S+E⟷C\to P+E, \end{array}$$

where *C* represents an intermediate complex formed by the action of the enzyme on the substrate. Let us for simplicity assume that the reaction rates are of the mass action type (with respective rate constants *k*_1_ and *k*_−1_ for the first reversible reaction, and *k*_2_ for the second reaction), then the mass balance model for this 2-step biochemical reaction network is:

$$\begin{array}{ccc} \frac{ds}{dt}\left(t\right) & = & -k_{1}es+k_{-1}c\\ \frac{de}{dt}\left(t\right) & = & -k_{1}es+k_{-1}c+k_{2}c\\ \frac{dc}{dt}\left(t\right) & = & k_{1}es-k_{-1}c-k_{2}c\\ \frac{dp}{dt}\left(t\right) & = & k_{2}c \end{array}$$

The key property for this network is the conservation of the following quantity:

$$\begin{array}{c} s(t)+e(t)+2c(t)+p(t), \end{array}$$

which is easily verified by showing that its derivative with respect to time is zero. If we integrate this biochemical reaction network in our chemostat model, then we obtain the following scaled chemostat model:

$$\begin{array}{ccc} \frac{ds}{dt}\left(t\right) & = & D\left(t\right)\left(S^{0}\left(t\right)-s\right)-k_{1}es+k_{-1}c \end{array}$$ (6)

$$\begin{array}{ccc} \frac{dp}{dt}\left(t\right) & = & k_{2}c-\left(x_{1}+x_{2}\right)f\left(p\right)-D\left(t\right)p \end{array}$$ (7)

$$\begin{array}{ccc} \frac{de}{dt}\left(t\right) & = & \left(1-q\right)x_{1}f\left(p\right)-k_{1}es+k_{-1}c+k_{2}c-D\left(t\right)e \end{array}$$ (8)

$$\begin{array}{ccc} \frac{dc}{dt}\left(t\right) & = & k_{1}es-k_{-1}c-k_{2}c-D\left(t\right)c \end{array}$$ (9)

$$\begin{array}{ccc} \frac{dx_{1}}{dt}\left(t\right) & = & x_{1}\left(qf\left(p\right)-D\left(t\right)\right) \end{array}$$ (10)

$$\begin{array}{ccc} \frac{dx_{2}}{dt}\left(t\right) & = & x_{2}\left(f\left(p\right)-D\left(t\right)\right) \end{array}$$ (11)

We show in S2 Appendix that the tragedy continues to hold, in the sense that the conclusion of Theorem 1 remains valid for this more general system.

Of course, more complicated biochemical reaction networks of the digestion process, with multiple intermediate complexes *C*_1_, …*C*_*n*_:

$$\begin{array}{c} S+E⟷C_{1}⟷\cdots ⟷C_{n}\to P+E \end{array}$$

could be used here instead, and the tragedy would continue to hold in such cases. The key property is that the mass balance equations corresponding to these networks should exhibit a conservation law to guarantee the boundedness of the solutions of the chemostat model which integrates this biochemistry. Most reasonable biochemical reaction networks do indeed possess such conservation laws.

### Cooperators can persist when cheaters are absent

We have shown that when cheaters are present initially, the total population of cooperators and cheaters, is doomed. Next we investigate what happens when cheaters are absent by considering a special case of the chemostat model (1)–(5) with *X*_2_ = 0, and constant operating parameters *D* and *S*^0^, which are both assumed to be positive:

$$\begin{array}{ccc} \frac{dS}{dt}\left(t\right) & = & D\left(S^{0}-S\right)-EG\left(S\right) \end{array}$$ (12)

$$\begin{array}{ccc} \frac{dP}{dt}\left(t\right) & = & EG\left(S\right)-\frac{1}{\gamma }X_{1}F\left(P\right)-DP \end{array}$$ (13)

$$\begin{array}{ccc} \frac{dE}{dt}\left(t\right) & = & \left(1-q\right)X_{1}F\left(P\right)-DE \end{array}$$ (14)

$$\begin{array}{ccc} \frac{dX_{1}}{dt}\left(t\right) & = & X_{1}\left(qF\left(P\right)-D\right) \end{array}$$ (15)

Notice that the nutrient processing rate has been specialized to *EG*(*S*), implying that it is proportional to the enzyme concentration *E*, and a possibly nonlinear function of the nutrient *G*(*S*). We replace assumption **H1**, by the following assumption, which introduces a monotonicity condition for *F*, and monotonicity and concavity condition for *G*:

$$\begin{array}{ccc} & H1’:G:R_{+}\to R_{+}\;\text{is}\;C^{2},G\left(0\right)=0,dG/dS\left(S\right)>0\;\text{for}\;\text{all}\;S>0,\;\text{and} & \\ & d^{2}G/dS^{2}\left(S\right)\leq 0\;\text{for}\;\text{all}\;S\geq 0,\;\text{and} & \\ & F:R_{+}\to R_{+}\;\text{is}\;C^{1},F\left(0\right)=0,dF/dP\left(P\right)>0\;\text{for}\;\text{all}\;P>0. \end{array}$$

The concavity condition for *G* will be used to limit the number of steady states of this system. The most commonly used choices for the functions for *F* and *G* are Monod functions (i.e. *F*(*P*) = *mP*/(*a* + *P*), where *a* and *m* are positive parameters), which satisfy these assumptions. But note that a linear function *G*(*S*) = *kS*, with *k* > 0 is allowed as well. In other words, the processing rate of nutrient (per unit of enzyme) does not necessarily have to saturate for large *S*-values.

The following dichotomy -global extinction, or bistability- is proved in S3 Appendix, and shows that the cooperator may persist when there are no cheaters; it refers to a scalar, nonlinear equation 23, which is given in S3 Appendix as well.

**Theorem 2** *Suppose that* **H1’** *holds, and that* $P^{*}:=F^{-1}\left(\frac{D}{q}\right)<S^{0}$.

1. *If equation 23 has no solutions, then the washout steady state* (0, 0, 0, 0) *is globally asymptotically stable for system* (12)–(15).
2. *If equation 23 has two distinct solutions, then system* (12)–(14) *has 3 steady states, the washout steady state* (0, 0, 0, 0) *and two positive steady states* *E*_1_ *and* *E*_2_. *The washout steady state and* *E*_2_ *are locally asymptotically stable, and* *E*_1_ *is a saddle with a three-dimensional stable manifold, and one-dimensional unstable manifold. The stable manifold is the common boundary of the regions of attraction of the washout steady state and* *E*_2_. *Every solution of system* (12)–(15) *converges to one of the three steady states. Persistence of the cooperator occurs for all initial conditions contained in the region of attraction of* *E*_2_, *and initial conditions on the stable manifold of the saddle* *E*_1_.

**Fig 3:**
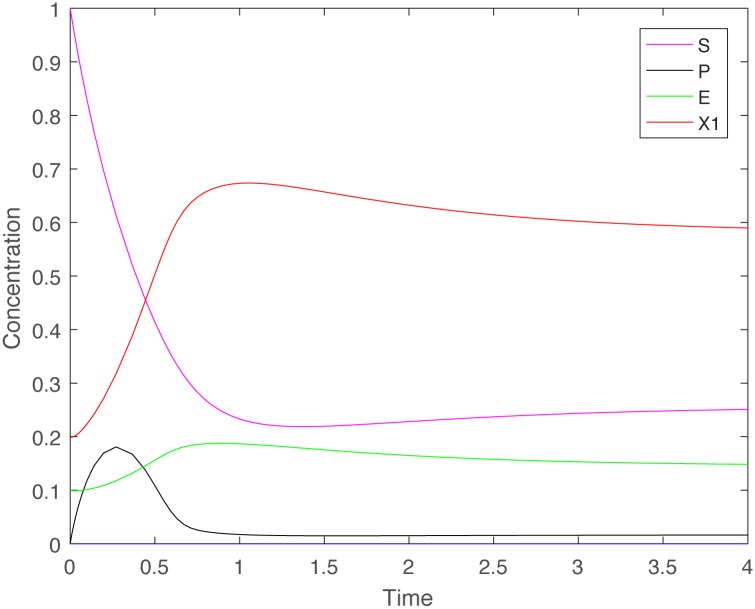
Time series of the cooperator population when cheaters are absent, illustrating cooperator persistence. Time series of the components of system (12)–(15), where *S*^0^ = 1, *D* = 1, *q* = 0.8, *γ* = 1, *G*(*E*, *S*) = *kES*, *F*(*P*) = *mP*/(*a* + *P*) with *k* = 20, *m* = 5 and *a* = 0.05. Initial data: *S*(0) = 1, *P*(0) = 0, *E*(0) = 0.1, *X*_1_(0) = 0.2.

Fig 3 illustrates the persistence of the cooperator in the absence of cheaters, even when there is no processed nutrient, and only a little amount of enzyme initially. Notice that the initial condition used in the simulation for Fig 3 is the same as the initial condition used for Fig 1, and the model parameters are the same as well. Nevertheless, the fate of the cooperator is very different: it goes extinct when the cheater is present initially (Fig 1), but persists otherwise (Fig 3).

## Discussion

Although the tragedy of the commons is such a pervasive notion in the recent developments of theories about the evolution of cooperation, we were unable to find any mathematical models that have rigorously analyzed an important group-level effect: the collapse of a population as a consequence of the dynamic interaction between cooperating and cheating individuals. The traditional approach to explain the tragedy has been to describe it in a game theoretical context, and attribute it to an *N*-person Prisoner’s Dilemma [32]. For a single run of a Prisoner’s Dilemma, the defection strategy which corresponds to cheating is a winning strategy and Nash equilibrium [33]. Repeated iterations of the Prisoner’s Dilemma enable the maintenance of cooperation, and various modifications have been proposed that build upon and extend this idea, including spatial effects [34, 35]. Some models combine game theory ideas with population growth models. In [36] for example, a Lotka-Volterra system of competing genotypes which produce none, or a mix of multiple public goods has been proposed. The cost of cooperation is modeled as a linear decrease of the intrinsic growth rate with respect to the number of public goods produced. Moreover, the carrying capacity is not fixed, but depends on the composition of the population. We note that this model does not explicitly model nutrients, nor the way in which the public goods chemically interact with the organisms, whereas these processes are explicitly modeled in our model. In another recent paper [37], the tradeoff between the population’s resilience to ecological perturbations that may induce population collapse via Allee effects, and its resistance to cheater invasion is investigated in the context of an Ecological Public Goods Game. Whereas low population numbers promote cooperative behavior, they may lead to population collapse due to ecological perturbations; on the other hand, high population numbers provide a buffer to ecological perturbations, but invite invasion by cheaters. Critical thresholds for the investment levels in cooperative behavior are determined in terms of the underlying public goods game which allow the population to optimize its behavior in the face of this tradeoff. In both of these cases, iterative cycles of population assembly, growth and dispersal are simulated. We do not consider any such perturbations in our model. Rather, we analyze and mathematically prove the existence of stable steady states.

While game theory can predict winning strategies, however, it generally does not consider the feedback of individual behavior phenotypes on group productivity. The main purpose of this paper was to offer an alternative, yet complementary approach to explain the tragedy that is not rooted in game theory and thereby avoids the explicit quantification of the payoffs of the various strategists, which appears to be particularly difficult for microbial populations. Our approach is purely mechanistic and our model merely expresses natural mass-balance equations. It incorporates substrate availability via intake from the feed bottle, production of the public good, the enzymatic conversion of substrate to product by the public good, uptake of the product, and cellular growth, and the washout of all chemical and biological compounds in the chemostat via dilution. All model parameters as well as functional forms can be quantified, determined and controlled experimentally, and there is no need to make abstract or ad hoc choices of payoffs. We have proved mathematically that the tragedy of the commons occurs in a chemostat system with cooperators that supply a public good required for growth, and cheaters that do not. The sole difference between cooperators and cheaters in this system is the cost associated with public good production, which is only experienced by the cooperator. While the cooperator diverts a fraction of the ingested nutrient from growth to public good production, the cheater invests everything in growth. We assume that there are no pleiotropic costs to cheating, and that the environment is well mixed, disregarding spatial structure as a major factor that promotes cooperation [6, 7]. Our results support the occurrence of the tragedy of the commons as a consequence of the selfish actions of individuals that result in the complete collapse of the shared public good [8, 17]. When this public good is essential for growth, the tragedy is manifested by the extinction of the whole group [14, 19, 23].

To understand how the tragedy of the commons arises in the chemostat, we perform a simple thought-experiment. Assume that initially there are no cheaters (*X*_2_(0) = 0), and suppose that the assumptions of Theorem 2 hold. If the initial condition of system (12)–(15) is contained in the region of attraction of the locally stable steady state *E*_2_, the solution will converge to, and eventually settles at this steady state. Numerical simulations (Fig 3) show that this can happen even if there is only a low initial amount of enzyme (*E*(0) is small), and no initial processed nutrient (*P*(0) = 0). The cooperator-only population therefore persists. However, if cheaters do suddenly appear -for example by mutation or by invasion into the environment- even in extremely low numbers, Theorem 1 shows that the total population of cooperators and cheaters is doomed, confirming the tragedy of the commons. One of the two proofs of Theorem 1 gives clues on how this happens: The *ratio of cooperators to cheaters* will always decrease. It may appear as if the cheaters will overtake the cooperators, and at least for a while, this is indeed what happens. However, in the long run there are not enough cooperators around to produce the enzyme levels required for nutrient processing, and this leads to the extinction of cheaters and cooperators alike.

To put our results in the context of Hardin’s original verbal description of the tragedy [8], we remark that Hardin did not explicitly distinguish between cooperators and cheaters, which is in contrast with recent interpretations of the tragedy in evolutionary biology [17]. In natural populations there are many different ways individuals can cooperate or cheat, and clearly articulating the distinction between both types is necessary to correlate it to the occurrence of the tragedy [17]. In its essence, the tragedy of the commons is the depletion of a common resource or a public good by the selfish action of competing individuals, thereby decreasing the average fitness of all individuals.

According to [17], the exploitation of different types of resources can give rise to a tragedy of the commons. The first, which fits Hardin’s analogy described above, involves the selfish exploitation of a common, extrinsic resource to the point of complete depletion, which causes all individuals to perish. The second type involves resources that are themselves the product of social behavior. In this case, the resource is a public good that is either formed by cooperation, or by restraining from conflict. Cooperation via public goods is pervasive in microbial social behavior, and it is also the case that we have described here with our model (1)–(5). As we have seen, the tragedy arises when non-cooperating cheaters reap the benefits provided by cooperators, without paying the costs. Microbial cooperative behaviors vulnerable to cheating include extracellular secretions such as enzymes and metabolites [14, 25, 38]. A particularly compelling example is the altruistic investment in the non-spore parts of a multicellular fruiting body in myxobacteria [18].

A different, more abstract, type of public good involves individuals restraining from potential conflict. A tragedy arises if the costs invested in compettitive behavior decrease overall productivity. In this case, less emphasis is placed on the depletion of extrinsic resources. A relevant example comes from another chemostat study which investigated the outcome of social conflict between different metabolic strategies in yeast, respiration and fermentation [38]. Respirers use glucose slowly but efficiently, whereas fermenters use glucose fast but wastefully. Thus, respiration is the strategy that provides the highest group-level benefit. Nevertheless, as shown experimentally and confirmed by simulation, fermenters are favored and fully displace respirers during glucose-limited growth in a chemostat [38]. Notably, in this system, as in restraint from conflict in general, one strategy does not obligately depend on the other for its success.

Our paper provides a simple paradigm of cheater-mediated population collapse. There are surprisingly few empirical reports of this phenomenon in the microbiological literature. To our knowledge, there is not a single example that employed a continuous culture system. It was therefore our intent to establish a null model for both experimentalists and theorists in which obligate cheating always causes population collapse.

Our results also have implications for biotechnological processes that rely on the cooperative behaviors among microbes for product synthesis, bioremediation and the treatment of wastewater. In these applications, the substrate *S* is considered to be unwanted, and the role of the microbes is to degrade it. They achieve this by producing an enzyme that targets the substrate for degradation into a form that they can use for their own growth. When all the cells cooperate and contribute to the production of the enzyme, this process can succeed (Theorem 2). But if an even minimal fraction of the cells cheat by not producing the enzyme, this process fails (Theorem 1): The microbial populations go extinct, and the unwanted substrate is not reduced.

As we have proven in this study, population collapse is inevitable in an obligate relationship, because the cooperator to cheater ratio always decreases. Eventually the cheater becomes so dominant that too little public good is produced by the cooperator, leading to the extinction of both types. The differential equation framework presented here will permit the in-depth analysis of mechanisms that promote cooperation. We have seen that if cooperator and cheater have the same yield, and the same per capita growth rate function, the tragedy is inevitable. This suggests that variations in yield constants and/or growth rates between cooperators and cheaters, which may arise via mutations, are necessary to avoid the tragedy in the chemostat. Future research will be conducted to assess if and when such changes do indeed promote cooperation. As mentioned earlier, spatial effects are known to sometimes promote the evolution of cooperation. Although the chemostat studied here is assumed to be well mixed and therefore does not include any spatial effects, it can be readily modified in ways similar to those described in Chapters 5 & 6, and Chapter 10 in [31], where space is incorporated discretely (gradostat), respectively continuously (unstirred chemostat). The results presented in this paper will serve as a benchmark to which the behavior of such spatially extended models can be compared in the pursuit of a deeper understanding of the mechanisms that promote cooperation in mechanistic models that do not rely on game theoretical ideas.

## Supporting information

S1 Appendix Proof of Theorem 1. (PDF)

S2 Appendix More general digestion networks. (PDF)

S3 Appendix Proof of Theorem 2. (PDF)
