# Supplementary material for: Tragedy of the commons in the chemostat
Source: PLoS One. 2017 Dec 20;12(12):e0186119. doi: 10.1371/journal.pone.0186119 (PMC5737893; doi:10.1371/journal.pone.0186119)
Supplement: S1 Appendix — (PDF) [file pone.0186119.s001.pdf]

S1 Appendix: Proof of Theorem 1

The model is:

$$\frac{dS}{dt}(t) = D(t)(S^0(t) - S) - G(E, S) \quad (1)$$

$$\frac{dP}{dt}(t) = G(E, S) - \frac{1}{\gamma}(X_1 + X_2) F(P) - D(t)P \quad (2)$$

$$\frac{dE}{dt}(t) = (1 - q)X_1 F(P) - D(t)E \quad (3)$$

$$\frac{dX_1}{dt}(t) = X_1 (qF(P) - D(t)) \quad (4)$$

$$\frac{dX_2}{dt}(t) = X_2 (F(P) - D(t)) \quad (5)$$

By scaling the state variables of system $(1) - (5)$ as follows:

$$\begin{aligned} s &= S \\ p &= P \\ e &= \frac{E}{\gamma} \\ x_1 &= \frac{X_1}{\gamma} \\ x_2 &= \frac{X_2}{\gamma}, \end{aligned}$$

and introducing the rescaled functions

$$\begin{aligned} g(e, s) &:= G(\gamma e, s) \\ f(p) &:= F(P), \end{aligned}$$

we obtain the following scaled model:

$$\frac{ds}{dt}(t) = D(t)(S^0(t) - s) - g(e, s) \quad (6)$$

$$\frac{dp}{dt}(t) = g(e, s) - (x_1 + x_2)f(p) - D(t)p \quad (7)$$

$$\frac{de}{dt}(t) = (1 - q)x_1 f(p) - D(t)e \quad (8)$$

$$\frac{dx_1}{dt}(t) = x_1 (qf(p) - D(t)) \quad (9)$$

$$\frac{dx_2}{dt}(t) = x_2 (f(p) - D(t)) \quad (10)$$

Notice that **H1**, which holds for the rate functions $G(E,S)$ and $F(P)$, is also valid for the scaled rate functions $g(e,s)$ and $f(p)$.

The total mass of this scaled model,

$$m = s + p + e + x_1 + x_2,$$

satisfies a linear equation:

$$\frac{dm}{dt}(t) = D(t)(S^0(t) - m), \tag{11}$$

which is easily verified by adding all the equations of the scaled model. This equation, and the upper bound for $S^0(t)$ in **H2** imply that the following family of compact sets

$$\Omega_\epsilon = \{(s,p,e,x_1,x_2)\,|\, s \geq 0, p \geq 0, e \geq 0, x_1 \geq 0, x_2 \geq 0, m \leq \bar{S}^0 + \epsilon\},$$

are forward invariant sets of the scaled model, for all $\epsilon \geq 0$.

The Main Result, Theorem 1, is an immediate Corollary of the following result, which is the tragedy of the commons for the scaled model:

Theorem 1. *Assume that* **H1** *and* **H2** *hold, and assume that the initial condition of* $(6)-(10)$ *is such that* $x_2(0) > 0$*; that is, the cheater is present initially. Then* $(p(t), e(t), x_1(t), x_2(t)) \to (0,0,0,0)$ *as* $t \to \infty$.

Proof

Given the initial condition, we can find an $\epsilon \geq 0$ such that the solution $(s(t), p(t), e(t), x_1(t), x_2(t))$ is contained in the compact set Ω_ϵ for all $t \geq 0$. We shall present two proofs. The first involves a (biologically nontrivial) transformation of one of the system's variables. The second considers the ratio of cooperators and cheaters, a biologically natural measure, and reveals that this ratio does not increase.

Proof 1: Consider the variable $y_2 = x_2^q$. Then

$$\frac{dy_2}{dt}(t) = y_2(qf(p) - qD(t))$$

Equation (9), and the above equation can be integrated:

$$\begin{aligned} x_1(t) &= x_1(0)\, \mathrm{e}^{\int_0^t qf(p(\tau)) - D(\tau) d\tau} \\ y_2(t) &= y_2(0)\, \mathrm{e}^{\int_0^t qf(p(\tau)) - qD(\tau) d\tau} > 0, \text{ for all } t \text{ since } y_2(0) = x_2^q(0) > 0, \end{aligned}$$

Dividing the first by the second equation yields:

$$x_1(t) = y_2(t)\frac{x_1(0)}{y_2(0)}\, \mathrm{e}^{-(1-q)\int_0^t D(\tau) d\tau} \leq B \frac{x_1(0)}{y_2(0)}\, \mathrm{e}^{-(1-q)\underline{D}t},$$

where we have used the lower bound for $D(t)$, see **H2**, to establish the last inequality, and the positive bound B for $y_2(t)$ which exists because the solution, and therefore also $x_2(t)$, is bounded. From this follows that $\lim_{t\to\infty} x_1(t) = 0$, where the

convergence is at least exponential with rate $(1-q)\underline{D}$.
Next we consider the dynamics of the variable $z = Qx_1 - e$, where $Q = (1-q)/q$:

$$\dot{z} = -D(t)z,$$

which is solvable, yielding $z(t) = z(0)\,\mathrm{e}^{-\int_0^t D(\tau)d\tau}$. The lower bound $\underline{D}$ for $D(t)$ in **H2**, then implies that $z(t) \to 0$ at a rate which is at least exponential with rate $\underline{D}$. This fact, together with the convergence of $x_1(t)$ to zero established above, implies that $e(t) \to 0$ as well.
Next, consider the p-equation (7). There holds that for each $\tilde{\epsilon} > 0$:

$$\frac{dp}{dt}(t) \leq \tilde{\epsilon} - \underline{\mathrm{D}}p, \text{ for all sufficiently large } t.$$

Notice that we used that $g(0,s) = 0$ for all $s \geq 0$, and the continuity of g, see **H1**, as well as **H2** for the lower bound of $D(t)$. It follows that $\limsup_{t\to\infty} p(t) \leq \tilde{\epsilon}/\underline{\mathrm{D}}$, and since $\tilde{\epsilon} > 0$ was arbitrary, there follows that $p(t) \to 0$.
Finally, we consider the x_2-equation (10). Since $p(t) \to 0$ and $f(0) = 0$ by **H1**, there holds that $f(p(t)) \leq \underline{\mathrm{D}}/2$ for all t sufficiently large. Consequently,

$$\frac{dx_2}{dt}(t) \leq -\frac{\underline{\mathrm{D}}}{2}x_2, \text{ for all sufficiently large } t,$$

and thus $x_2(t) \to 0$, concluding the proof in this case.

Proof 2: Equations (9) and (10) can be integrated:

$$\begin{aligned} x_1(t) &= x_1(0)\,\mathrm{e}^{\int_0^t qf(p(\tau))-D(\tau)d\tau} && (12) \\ x_2(t) &= x_2(0)\,\mathrm{e}^{\int_0^t f(p(\tau))-D(\tau)d\tau} > 0, \text{ for all } t \text{ since } x_2(0) > 0. && (13) \end{aligned}$$

Thus, the ratio $r(t) = x_1(t)/x_2(t)$ is well-defined and satisfies the differential equation:

$$\frac{dr}{dt}(t) = -(1-q)f(p)r,$$

which shows that the ratio does not increase. The solution of this equation is:

$$r(t) = r(0)\,\mathrm{e}^{-(1-q)\int_0^t f(p(\tau))d\tau} \qquad (14)$$

We distinguish two cases depending on the integrability of the function $f(p(t))$:

Case 1: $\int_0^\infty f(p(\tau))d\tau = \infty$.
It follows from (14) that $r(t) \to 0$, and hence also $x_1(t) \to 0$ because $x_2(t)$ is bounded. Proof of convergence of $e(t), p(t)$ and $x_2(t)$ to zero now proceeds as in **Proof 1**.

Case 2: $\int_0^\infty f(p(\tau))d\tau < \infty$.
It follows from $(12)-(13)$ that both $x_1(t) \to 0$ and $x_2(t) \to 0$, because $0 < \underline{D} \leq D(t)$ for all t, by **H2**. Proof of convergence of $e(t)$ and $p(t)$ to zero now proceeds as in **Proof 1** as well.
