# Supplementary material for: Tragedy of the commons in the chemostat
Source: PLoS One. 2017 Dec 20;12(12):e0186119. doi: 10.1371/journal.pone.0186119 (PMC5737893; doi:10.1371/journal.pone.0186119)
Supplement: S2 Appendix — (PDF) [file pone.0186119.s002.pdf]

S2 Appendix: More general digestion networks

To see why the tragedy continues to hold for more general digestion network, we consider solutions of system the following system:

$$\frac{ds}{dt}(t) = D(t)(S^0(t) - s) - k_1 es + k_{-1}c \tag{1}$$
$$\frac{dp}{dt}(t) = k_2 c - (x_1 + x_2)\, f(p) - D(t)p \tag{2}$$
$$\frac{de}{dt}(t) = (1-q)x_1 f(p) - k_1 es + k_{-1}c + k_2 c - D(t)e \tag{3}$$
$$\frac{dc}{dt}(t) = k_1 es - k_{-1}c - k_2 c - D(t)c \tag{4}$$
$$\frac{dx_1}{dt}(t) = x_1\,(qf(p) - D(t)) \tag{5}$$
$$\frac{dx_2}{dt}(t) = x_2\,(f(p) - D(t))\,, \tag{6}$$

for which it is easily verified that the variable:

$$m = s + p + e + 2c + x_1 + x_2,$$

still satisfies equation

$$\frac{dm}{dt}(t) = D(t)(S^0(t) - m), \tag{7}$$

implying that the family of compact sets Ω_ϵ, defined earlier, is forward invariant for system (1) – (6), for all $\epsilon \geq 0$, when **H2** holds. Consequently, the proof of Theorem 1 in S4 remains valid for the above chemostat model (1) – (6). Indeed, the first proof only crucially depends on the dynamics of x_1 and x_2 to show that $x_1(t)$ converges to zero, after which the convergence of e, p and x_2 is obtained by elementary comparison arguments. For the digestion network presented here, the dynamics of x_1 and x_2 remain unchanged, hence we can still conclude that $x_1(t)$ converges to zero. After that, it follows from a comparison argument that $e + c$ converges to zero, and then similarly that p and x_2 converge to zero as well. One could also easily adapt the steps of the second proof to obtain the same conclusion.
