# Supplementary material for: Tragedy of the commons in the chemostat
Source: PLoS One. 2017 Dec 20;12(12):e0186119. doi: 10.1371/journal.pone.0186119 (PMC5737893; doi:10.1371/journal.pone.0186119)
Supplement: S3 Appendix — (PDF) [file pone.0186119.s003.pdf]

S3 Appendix: Proof of Theorem 2

The model is:

$$\frac{dS}{dt}(t) = D(S^0 - S) - EG(S) \quad (1)$$

$$\frac{dP}{dt}(t) = EG(S) - \frac{1}{\gamma}X_1F(P) - DP \quad (2)$$

$$\frac{dE}{dt}(t) = (1-q)X_1F(P) - DE \quad (3)$$

$$\frac{dX_1}{dt}(t) = X_1\left(qF(P) - D\right) \quad (4)$$

By scaling the state variables of system $(1) - (4)$ in the usual way as follows:

$$s = S$$
$$p = P$$
$$e = \frac{E}{\gamma}$$
$$x_1 = \frac{X_1}{\gamma},$$

and switching to lower case letters for the rate functions:

$$g(s) := G(S)$$
$$f(p) := F(P),$$

and for the chemostat's constant operating parameters:

$$d := D$$
$$s^0 := S^0,$$

we obtain the following scaled model:

$$\frac{ds}{dt}(t) = d(s^0 - s) - eg(s) \quad (5)$$

$$\frac{dp}{dt}(t) = eg(s) - x_1f(p) - dp \quad (6)$$

$$\frac{de}{dt}(t) = (1-q)x_1f(p) - de \quad (7)$$

$$\frac{dx_1}{dt}(t) = x_1\left(qf(p) - d\right) \quad (8)$$

Notice that **H1'**, which holds for the rate functions $G(S)$ and $F(P)$, is also valid for the rate functions $g(s)$ and $f(p)$.

We introduce two new variables:

$$m = s+p+e+x_1 \tag{9}$$

$$v = e - Qx_1, \text{ where } Q := \frac{1-q}{q} \tag{10}$$

and choose to drop the s and e-equations from system (5) – (8), transforming it to:

$$\frac{dm}{dt}(t) = d(s^0 - m) \tag{11}$$

$$\frac{dv}{dt}(t) = -dv \tag{12}$$

$$\frac{dp}{dt}(t) = (v+Qx_1)g(m-p-v-x_1/q) - xf(p) - dp \tag{13}$$

$$\frac{dx_1}{dt}(t) = x_1\,(qf(p)-d) \tag{14}$$

with state space $\{p, x_1 \geq 0 \,:\, m \geq p+v+x_1/q,\ v+Qx_1 \geq 0\}$, which is forward invariant. The variables $m(t)$ and $v(t)$ converge exponentially to s^0 and 0 respectively, hence it is natural to study the limiting system:

$$\frac{dp}{dt}(t) = Qx_1 g(s^0-p-x_1/q) - x_1 f(p) - dp \tag{15}$$

$$\frac{dx_1}{dt}(t) = x_1(qf(p)-d) \tag{16}$$

which is defined on the state space $\{p, x_1 \geq 0 \,:\, p+x_1/q \leq s^0\}$, which is forward invariant. It turns out to be more convenient to transform this system using the variable:

$$w = p + \frac{1}{q}x_1, \tag{17}$$

instead of the variable p, yielding:

$$\frac{dw}{dt}(t) = Qx_1 g(s^0-w) - dw \tag{18}$$

$$\frac{dx_1}{dt}(t) = x_1(qf(w-x_1/q)-d) \tag{19}$$

with state space $\Omega_{red} = \{x_1 \geq 0 :\ x_1/q \leq w \leq s^0\}$, which is forward invariant.

We start our analysis of system (18) – (19) by determining the nullclines. The w-nullcline is given by:

$$x_1 = h(w), \text{ where } h(w) = \frac{d}{Q}\frac{w}{g(s^0-w)}. \tag{20}$$

The main properties of the function $h(w) : [0, s^0) \to \mathbb{R}_+$ are:

1. $h(0) = 0$, and $\lim_{w\to s^0} h(w) = \infty$.
2. $h'(w) = \frac{d}{Q}\frac{g(s^0-w)+wg'(s^0-w)}{g^2(s^0-w)} > 0$, by **H1'**.

3. $h''(w) = \frac{d}{Q} \frac{-wg''(s^0-w)g^2(s^0-w)+2g(s^0-w)g'(s^0-w)\left(g(s^0-w)+wg'(s^0-w)\right)}{g^4(s^0-w)} > 0$, by **H1′**.

Thus, the function $h(w)$ is zero at zero, is increasing with a vertical asymptote at $w = s^0$, and it is strictly convex.

To obtain a nontrivial x_1-nullcline in the state space, we make one more assumption, namely:

$$p^* := f^{-1}\left(\frac{d}{q}\right) \text{ satisfies } p^* < s^0. \tag{21}$$

This assumption merely expresses that the cooperator has a break-even steady state concentration for the processed nutrient at a level below the input nutrient concentration s^0, see equation (8). In addition to the horizontal axis $x_1 = 0$, there is a nontrivial x_1-nullcline which is particularly easy to express using p^*, as the graph of a linear function:

$$x_1 = q(w - p^*). \tag{22}$$

Any nonzero steady states of the limiting system are given by the intersection of the w- and the nontrivial x_1-nullcine, which are determined by the solutions of the equation:

$$h(w) = q(w - p^*),\ 0 \le w < s^0. \tag{23}$$

In view of the convexity of the function h, there are either no, one, or two solutions to (23), and generically there are none, or two. We will construct the phase portrait of the limiting system in these two cases.

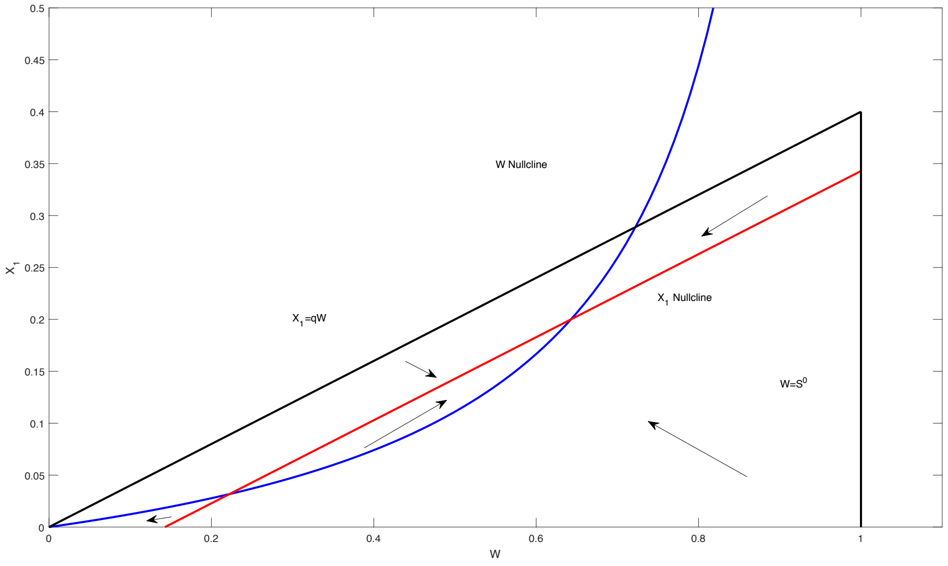

Figure 1: Phase plane of the limiting system (18) − (19) in case there are two nonzero steady states.

Lemma 1. *Suppose that* **H1'** *and* (21) *hold.*

1. *If equation* (23) *has no solutions, then system* $(18)-(19)$ *has a unique steady state* $(0,0)$ *which is globally asymptotically stable with respect to initial conditions in* Ω_{red}.

2. *If equation* (23) *has two solutions* $w_1 < w_2$, *then system* $(18)-(19)$ *has 3 steady states,* $(0,0)$, $(w_1, h(w_1))$ *and* $(w_2, h(w_2))$. *The steady states* $(0,0)$ *and* $(w_2, h(w_2))$ *are locally asymptotically stable, and* $(w_1, h(w_1))$ *is a saddle with one-dimensional stable manifold* W_s, *and one-dimensional unstable manifold* W_u. *The stable manifold* W_s *intersects the boundary of* Ω_{red} *in two points, one on the boundary* $x_1 = qw$, *the other on the boundary* $w = s^0$, *forming a separatrix: Initial conditions below* W_s *give rise to solutions converging to* $(0,0)$, *whereas initial conditions above* W_s *give rise to solutions converging to* $(w_2, h(w_2))$, *yielding bistability in the limiting system, see Fig* 1.

Proof

1. If (23) has no solutions, then the w-nullcline and the nontrivial x_1-nullcline do not intersect, and thus $(0,0)$ is the only steady state of the system. The state space Ω_{red} is divided in 3 parts by the two nullclines, and it is easy to see that the region enclosed between both nullclines and the boundary of Ω_{red} is a trapping region in which solutions monotonically converge to the zero steady state. Solutions starting in the region above the w-nullcline are monotonically decreasing (increasing) in the x_1-component (w-component), but since that region does not contain nontrivial steady states, these solutions must enter the trapping region between both nullclines. Similarly, solutions that start below the x_1-nullcline are monotonically increasing (decreasing) in the x_1-component (w-component), and must enter the trapping region as well. This concludes the proof of the assertion that $(0,0)$ is a globally asymptotically stable steady state.

2. If equation (23) has two solutions $w_1 < w_2$, then the nullclines intersect in two distinct points, yielding the positive steady states $(w_1, h(w_1))$ and $(w_2, h(w_2))$, see Fig 1. The third steady state is $(0,0)$. It is not hard to see that the state space is now divided into 5 parts, 3 of which are trapping regions. Each of these trapping regions is enclosed by arcs of the nullclines or segments of the boundary of Ω_{red} which either connect pairs of steady states, or a steady state and a point on the boundary of the state space Ω_{red}, see Fig 1. There are also 2 remaining regions, which we call the NW and SE regions, for obvious reasons.

   To complete the phase plane analysis, we perform a linearization of the system at the steady states. The Jacobian matrix of the limiting system is

   $$\begin{pmatrix} -Qx_1 g'(s^0 - w) - d & Qg(s^0 - w) \\ x_1 q f'(w - x_1/q) & (qf(w - x_1/q) - d) - x_1 f'(w - x_1/q) \end{pmatrix}$$

   We focus on the middle steady state $(w_1, h(w_1))$, where the Jacobian evaluates to:

   $$J_1 = \begin{pmatrix} -d\left(\frac{w_1 g'(s^0 - w_1)}{g(s^0 - w_1)} + 1\right) & Qg(s^0 - w) \\ \frac{d}{Q} q \frac{w_1}{g(s^0 - w_1)} f'(p^*) & -\frac{d}{Q}\frac{w_1}{g(s^0 - w_1)} f'(p^*) \end{pmatrix}$$

Clearly, the trace is negative, and the determinant is given by:

$$\frac{d}{Q}\frac{w_1}{g(s^0-w_1)}f'(p^*)\left[d\left(\frac{w_1 g'(s^0-w_1)}{g(s^0-w_1)}+1\right)-qQg(s^0-w_1)\right]$$

We claim that this determinant is negative, which implies that this steady state is a saddle. This follows from the fact that the slope of the tangent line to the graph of the convex function $h(w)$ at $w=w_1$ must be smaller than the slope of the line $x_1=q(w-p^*)$, which is of course q:

$$h'(w_1)<q.$$

Recalling the derivative of $h(w)$ given above, it can be shown that this latter inequality is equivalent to the expression in the square brackets in the determinant being negative, which establishes the claim.

Incidentally, a similar argument can be used to show that the determinant of the linearization at the steady state $(w_2,h(w_2))$ is positive, because in this case $h'(w_2)>q$. Since the trace of that linearization is also negative, this shows that $(w_2,h(w_2))$ is locally asymptotically stable. The linearization at $(0,0)$ is triangular with both diagonal entries equal to $-d$, from which also follows that $(0,0)$ is locally asymptotically stable.

Now we turn to the question of the location of the one-dimensional stable and unstable manifolds W_s, respectively W_u, of the saddle. Therefore, we determine the eigenvectors of the negative eigenvalue λ_1, and the positive eigenvalue λ_2 of the Jacobian matrix J_1. We have that

$$J_1\begin{pmatrix}1\\r_1\end{pmatrix}=\lambda_1\begin{pmatrix}1\\r_1\end{pmatrix}\text{ with }\lambda_1<0,\text{ and }J_1\begin{pmatrix}1\\r_2\end{pmatrix}=\lambda_2\begin{pmatrix}1\\r_2\end{pmatrix},\text{ with }\lambda_2>0,$$

where we wish to determine, or at least estimate, r_1 and r_2. We can find r_1 by considering the first of the two equations determining λ_1:

$$r_1=\frac{1}{g(s^0-w)}\left(\lambda_1+d\left(\frac{w_1 g'(s^0-w_1)}{g(s^0-w_1)}+1\right)\right).$$

We claim that $r_1<0$. Indeed, since the trace of J_1 (which equals $\lambda_1+\lambda_2$) is less than the top-left entry of J_1, it follows that the expression in the large parentheses is less than $-\lambda_2$, which is negative. This implies that near $(w_1,h(w_1))$, the stable manifold has a branch in the NW, and another branch in the SE region. Backward integration of solutions starting near the saddle and on W_s in these regions, shows that they must either exit these regions along the boundary of Ω_{red} (because backward-time solutions cannot exit via the trapping regions), or they must converge to a steady state. However, there are no steady states to the NW of the saddle in the NW region, nor to the SE of the saddle in the SE region. Thus, the stable manifold W_s must intersect the boundary of Ω_{red} in two points, one on the line $x_1=qw$, and the other on the line $w=s^0$. Next, we focus on the location of the unstable manifold W_u of the saddle, whose location is determined by r_2. We claim that:

$$h'(w_1)<r_2<q.$$

To see this we consider the first equation in the eigenvalue equation for λ_2, by solving for r_2, once again recalling the expression of the derivative of the function h:

$$r_2 = \frac{\lambda_2}{Qg(s^0 - w_1)} + h'(w_1),$$

from which the first inequality follows because $\lambda_2 > 0$. The second equation in the eigenvalue equation yields that:

$$r_2 = q\frac{h(w_1)f'(p^*)}{h(w_1)f'(p^*) + \lambda_2},$$

and again, since $\lambda_2 > 0$, we find that the second inequality holds, as claimed.

We can now fully assemble the phase portrait presented in Fig 1. The stable manifold W_s of the saddle has a branch in the first and second trapping regions. Solutions starting on these branches must converge to $(0,0)$, and $(w_2, h(w_2))$ respectively. In fact, it is not hard to see that all solutions in the first trapping region converge to $(0,0)$, whereas solutions in the second and third trapping region converge to $(w_2, h(w_2))$. The fate of solutions starting in the NW and SE regions depends on their initial location relative to the separatrix W_s: They converge to $(0,0)$ if they start below W_s, but to $(w_2, h(w_2))$ if they start above W_s, and this occurs because they must enter one of the trapping regions first.

The asymptotic behavior of the solutions of system (18) − (19) described in Lemma 1 can be translated into the asymptotic behavior of the solutions of system (15) − (16), and combining this with the theory of asymptotically autonomous systems, see Appendix F in [1], the asymptotic behavior of the transformed system (11) − (14) can be obtained as well. In turn, this determines the behavior of the scaled system (5) − (8), from which Theorem 2 follows immediately.

References

[1] H.L. Smith, and P. Waltman, The Theory of the Chemostat (Dynamics of Microbial Competition), Cambridge University Press, 1995.
